# The fragile X mental retardation protein regulates tumor invasiveness-related pathways in melanoma cells

**DOI:** 10.1038/cddis.2017.521

**Published:** 2017-11-16

**Authors:** Francesca Zalfa, Vincenzo Panasiti, Simone Carotti, Maria Zingariello, Giuseppe Perrone, Laura Sancillo, Laura Pacini, Flavie Luciani, Vincenzo Roberti, Silvia D'Amico, Rosa Coppola, Simona Osella Abate, Rosa Alba Rana, Anastasia De Luca, Mark Fiers, Valentina Melocchi, Fabrizio Bianchi, Maria Giulia Farace, Tilmann Achsel, Jean-Christophe Marine, Sergio Morini, Claudia Bagni

**Affiliations:** 1 Department of Medicine, Campus Bio-Medico University, via Alvaro del Portillo 21, 00128 Rome, Italy; 2 Department of Medicine and Science of Aging, University of Chieti 'G d’Annunzio', via dei Vestini 31, 66100 Chieti-Pescara, Italy; 3 Department of Biomedicine and Prevention, University of Rome 'Tor Vergata', via Montpellier 1, 00133 Rome, Italy; 4 VIB/Center for the Biology of Disease, KU Leuven, O&N 4, Herestraat 49 Box 602, 3000, Leuven, Belgium; 5 Center for Human Genetics, Leuven Institute for Neuroscience and Disease, KU Leuven, O&N 4, Herestraat 49 Box 602, Leuven, 3000, Belgium; 6 Department of Dermatology, University of Rome 'La Sapienza', viale dell’Università 1, 00185 Rome, Italy; 7 Department of Medical Science and Human Oncology, Section of Dermato-Oncology, University of Turin, via Verdi 8, 10124 Turin, Italy; 8 ISBREMIT, Institute for Stem-cell Biology, Regenerative Medicine and Innovative Therapies, IRCCS Casa Sollievo della Sofferenza, viale Padre Pio 7, 71013 San Giovanni Rotondo (FG), Italy; 9 Department of Fundamental Neuroscience, University of Lausanne, Rue du Bugnon 9, 1005 Lausanne, Switzerland

## Abstract

The fragile X mental retardation protein (FMRP) is lacking or mutated in patients with the fragile X syndrome (FXS), the most frequent form of inherited intellectual disability. FMRP affects metastasis formation in a mouse model for breast cancer. Here we show that FMRP is overexpressed in human melanoma with high Breslow thickness and high Clark level. Furthermore, meta-analysis of the TCGA melanoma data revealed that high levels of FMRP expression correlate significantly with metastatic tumor tissues, risk of relapsing and disease-free survival. Reduction of FMRP in metastatic melanoma cell lines impinges on cell migration, invasion and adhesion. Next-generation sequencing in human melanoma cells revealed that FMRP regulates a large number of mRNAs involved in relevant processes of melanoma progression. Our findings suggest an association between FMRP levels and the invasive phenotype in melanoma and might open new avenues towards the discovery of novel therapeutic targets.

Mutations or absence of FMRP cause the fragile X syndrome (FXS), the most frequent form of inherited intellectual disability in humans.^1^ FMRP is a RNA-binding protein (RBP) involved in multiple steps of RNA metabolism. In the brain, its functional absence causes impaired synaptic plasticity due to defects in cytoskeletal organization and receptor mobility at synapses.^1, 2, 3^ Specifically, FMRP can act as a negative regulator of translation,^1, 4, 5, 6^ modulate the stability of RNA messengers,^7, 8, 9, 10^ regulate mRNA transport^11, 12^ or affect RNA editing^13, 14^ depending on the identity of the target mRNA, the presence of noncoding RNAs and the cellular context. Of note, FMRP-regulated mRNAs are involved in cytoskeleton remodeling and cell adhesion, mechanisms also involved in cancer progression and metastatization.^15, 16^

Converging evidence from a limited number of studies highlight the involvement (direct or indirect) of FMRP in cancer: (1) the gene *FMR1*, encoding for FMRP, is expressed in different tissues and cancer cell types (https://www.genevestigator.com/gv/); (2) individuals with FXS have a documented decreased risk of cancer;^17, 18^ (3) high levels of FMRP are linked to metastatic breast cancer;^18^ (4) *FMR1* mRNA is overexpressed in hepatocellular carcinoma cells;^19, 20^ (5) a reduced glioblastoma invasiveness has been reported in a patient with FXS;^21^ (6) the *FMR1* autosomal paralog and interactor, *FXR1*, was recently identified as a predictor of distant metastasis in triple-negative breast cancer;^22^ (7) several FMRP mRNA targets are involved in cancer progression.^23^

In the present work, we investigated the role of FMRP in melanoma, a neoplasm that accounts for ~75% of all deaths due to skin cancer.^24, 25^ A critical step in melanoma progression seems to be the transition from radial to vertical growth phase.^26^ This switch is associated with molecular and genetic changes that facilitate metastatization.^27, 28^ However, the molecular mechanisms that mediate differential expression of genes during melanoma progression remain largely unknown. Here we show that FMRP is overexpressed in human melanomas characterized by high Breslow thickness and high Clark level. We found that FMRP levels correlate with prognostic factors of aggressive melanoma and FMRP is often detected at high levels in cells localized at invasive front of the tumor. Moreover, a meta-analysis of The Cancer Genome Atlas (TCGA) melanoma data set revealed that increased *FMR1* expression level significantly correlates with metastatic melanoma, risk of tumor relapse and reduced disease-free survival. Reduction of FMRP in two melanoma cell lines revealed decreased cellular migration and invasion and increased adhesion properties. Finally, using next-generation sequencing, we identified the FMRP-regulated transcriptome in melanoma cells. Gene Ontology (GO) and Kyoto Encyclopedia of Genes and Genomes (KEGG) databases revealed that FMRP affects gene expression of almost 300 proteins involved in invasiveness-related pathways. Our findings suggest that FMRP could affect melanoma progression through the action of proteins involved in plasma membrane plasticity at the leading edges of cancer cells, driving their invasiveness.

## Results

### FMRP is highly expressed in human melanoma

FMRP expression was analyzed by IHC with a specific FMRP antibody,^29^ in a panel of formalin-fixed paraffin-embedded tumor tissues (*N*=64) classified into four main subtypes: lentigo maligna melanoma (LMM), superficial spreading melanoma (SSM), acral lentiginous melanoma (ALM) and nodular melanoma (NM), the only histotype showing vertical growth phase *ab initio*.

**Figure 1:**
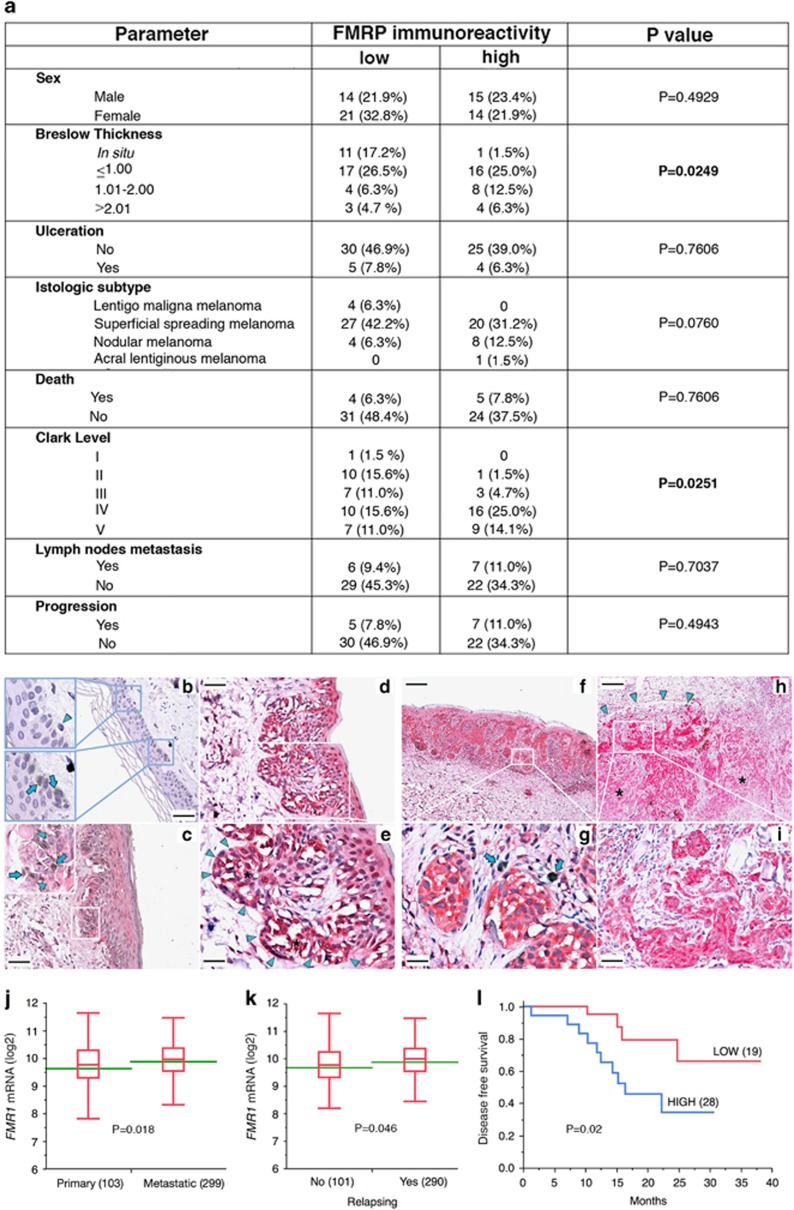
FMRP is highly expressed in human melanoma with higher Breslow thickness. (**a**) Correlation between FMRP immunoreactivity and different parameters analyzed in a cohort (*n*=64) of human melanoma. FMRP-specific antibodies were described in Luca *et al.*^18^ and Ferrari *et al.*^29^ The number of cases with weak-moderate FMRP level (FMRP-IHC≤1) and high level of FMRP (FMRP-IHC>1), as well as the percentage of FMRP-positive cases (%) is reported in each patient subgroup together with a summary of clinical–pathological parameters. FMRP correlated with high Breslow thickness and high Clark level (*P*=0.0249 and *P*=0.0251, respectively). (**b-i**) Representative images of FMRP immunoreactivity in normal skin and in three different histological subtypes of melanoma. FMRP immunoreactivity was low in melanocytes from normal skin (arrowheads) (b). FMRP-positive neoplastic cells (arrowheads) were observed in *in situ* melanoma (ISM) (c), SSM (d-g) and NM (h and i), and where the higher Breslow index was observed, the higher level of FMRP expression was found. Breslow (d and e)=0.3 mm; Breslow (f and g)=0.69 mm; Breslow (h and i)=5 mm. Increased FMRP positivity was frequently found at the periphery of neoplastic nests in SSM (d and e, high power field, arrowheads) and at the invasive front in NM (arrowheads, h and i, high power field), compared with other tumoral zones (asterisks). Arrows: Azure B-positive melanin granules. Original magnification: b, c and d, × 200, calibration bar 50 *μ*m; f and h × 40, calibration bar 250 *μ*m; high power fields: e and g × 400, calibration bar 25 *μ*m; i × 200, calibration bar 50 *μ*m. Counterstains: Hematoxylin and Azure B. (**j-l**) *FMR1* mRNA expression in the skin cutaneous melanoma TCGA data set and Kaplan–Meier curves. (**j**), *FMR1* mRNA expression analysis in primary melanoma samples and in metastatic melanoma. Box plots indicate the distribution of log 2 *FMR1* mRNA expression in the two classes. Green lines represent the average *FMR1* mRNA expression. *P*=0.018, Student's *t*-test. Within parentheses are the number of samples in each class. (**k**) *FMR1* mRNA expression analysis in tumors that relapse after initial treatment (YES) or not (NO). Box plots indicate the distribution of log 2 *FMR1* mRNA expression in the two classes, and green lines represent the average expression. *P*=0.046, Student's *t*-test. Within parentheses are the number of samples in each class. (**l**) Kaplan–Meier plot of patients with melanoma stratified by *FMR1* mRNA expression level in the primary tumor (TCGA skin cutaneous melanoma data). Probability of disease-free survival (DFS) is shown for the two categories (*FMR1* high and low; see Materials and Methods). Within parentheses are the number of patients in each category. *P*=0.02, Student's *t*-test

FMRP was significantly overexpressed in melanomas with higher Breslow thickness (Figure 1a; *P*=0.0249, *χ*^2^ test). Furthermore, FMRP levels correlated with high Clark level (Figure 1a, *P*=0.0251, *χ*^2^ test). Histopathological analysis of primary tumors revealed that FMRP immunoreactivity was low in melanocytes from normal skin (Figure 1b, arrowheads), but increased progressively in *in situ* melanoma (Figure 1c, arrowheads), SSM (Figure 1d and g) and NM (Figure 1h and i). Importantly, increased FMRP positivity was frequently found at the periphery of neoplastic nests in SSM (Figure 1d and e, high power field, arrowheads) and a marked expression of FMRP was detected in the cells at the invasive front of NM (Figure 1h and i, high power field, arrowheads). These observations suggest that cancer cells with increased FMRP expression are more likely to acquire the ability to leave the primary tumor, giving rise to distant metastases. Accordingly, an *in silico* analysis of a melanoma cohort (402 patients) from publicly accessible TCGA data set (RNA-sequence (RNA-seq) data) showed that increased *FMR1* mRNA expression level significantly correlated with metastatic melanoma (Figure 1j) and risk of tumor relapse (Figure 1k). Moreover, a survival analysis, comparing high- (Figure 1j) and low-expressing *FMR1* primary melanoma (*N*=47), showed a significant decreased disease-free survival in patients with *FMR1*-overexpressing tumors (Figure 1l).

**Figure 2:**
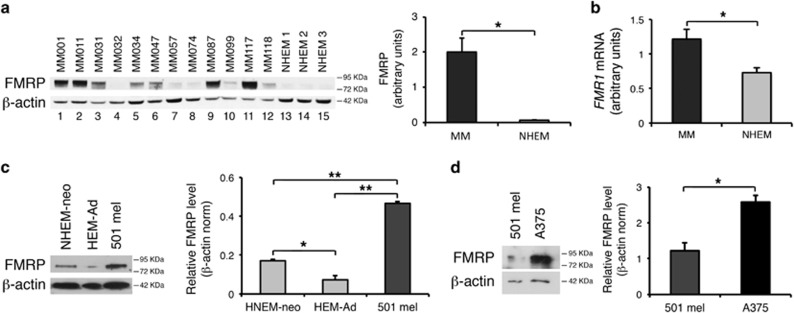
FMRP is highly expressed in metastatic melanoma. (**a**, Left) Representative FMRP levels analyzed by western blotting using specific FMRP antibodies^29^ in 12 cell lines from patients with invasive metastatic melanomas (MM, lanes 1–12) and in 3 cell lines of NHEMs (lanes 13–15). (Right) Quantification upon normalization for *β*-actin (**b**) *FMR1* mRNA levels in the same samples as in (**a**). (**c**, Left) Representative western blotting of FMRP levels in NHEM-neo, HEM-Ad and melanoma cell line 501 mel. (Right) protein levels are quantified upon normalization for *β*-actin. (**d**, Left) Representative western blotting of FMRP levels in 501 mel and A375 cell lines. (Right) Quantification upon normalization for *β*-actin (*n*=3, *P<0.05, **P<0.01, Student's *t*-test)

FMRP expression was also analyzed by semiquantitative western blotting of 12 melanoma cell lines derived from patients with invasive and metastatic melanomas (MM), upon being shortly cultured (melanoma short-term cultures).^30^ The overall expression of FMRP was significantly increased in melanoma cells compared with normal human epidermal melanocytes (NHEMs) (Figure 2a). Consistently, *FMR1* mRNA expression was increased in MM cells compared with NHEM (Figure 2b). We further investigated the expression of FMRP in two metastatic melanoma cell lines, the pigmented 501 mel^31^ and the unpigmented A375.^32, 33^ The 501 mel cell line exhibited higher FMRP levels compared with control adult human epidermal melanocytes (HEM-Ad) and neonatal NHEMs (NHEM-neo) (Figure 2c). Of note, NHEM-neo melanocytes expressed higher levels of FMRP compared with HEM-Ad (Figure 2c), suggesting that, as in the brain,^34, 35^ FMRP expression might be regulated during skin development. Finally, the A375 cell line had higher FMRP levels compared with the 501 mel (Figure 2d). Overall, these findings suggest that FMRP overexpression is associated with melanoma progression, and particularly the metastatic phenotype.

### FMRP levels affect migration, invasion and adhesion in human melanoma cells

To verify whether lack of FMRP might lead to different capacity of melanoma cells to migrate, invade and/or adhere, we knocked down FMRP expression in 501 mel (Supplementary Figure 1a) or A375 cells (Supplementary Figure 1b), treating the cells for 24, 48 or 72 h with a scrambled or specific small interfering RNAs (siRNAs) (*FMR1* siRNA). In both cell lines, FMRP was nearly silenced 72 h after siRNA transfection (Supplementary Figures 1a and b) and remained silenced up to 6 days after siRNA treatment (data not shown).

**Figure 3:**
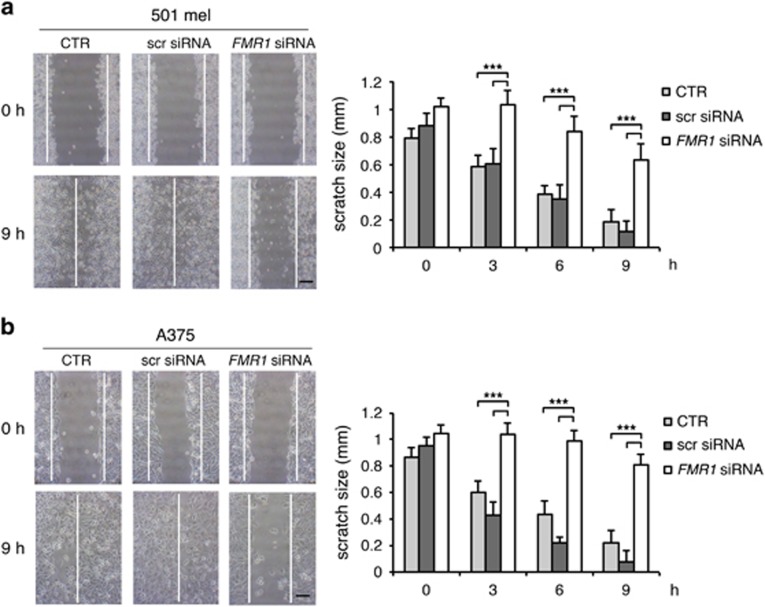
Downregulation of FMRP in melanoma cells affects migration. (**a**) Wound-healing assay in 501 mel cells treated with *FMR1* siRNA, with scrambled siRNA (scr siRNA) or untransfected cells (CTR). (Left panel) A single scratch was made in the center of cell monolayer and the wound closure areas visualized under an inverted microscope with a x20 magnification. (Right panel) Cell motility was quantified by measuring the distance between the invading front of cells in 10 random selected microscopic fields for each single condition and time point (right panel) (*n*=3, ***P<0.001, Student's *t*-test). (**b**) Same as in panel a for the A375 cell line. Calibration bars 250 *μ*m in (**a** and **b**)

**Figure 4:**
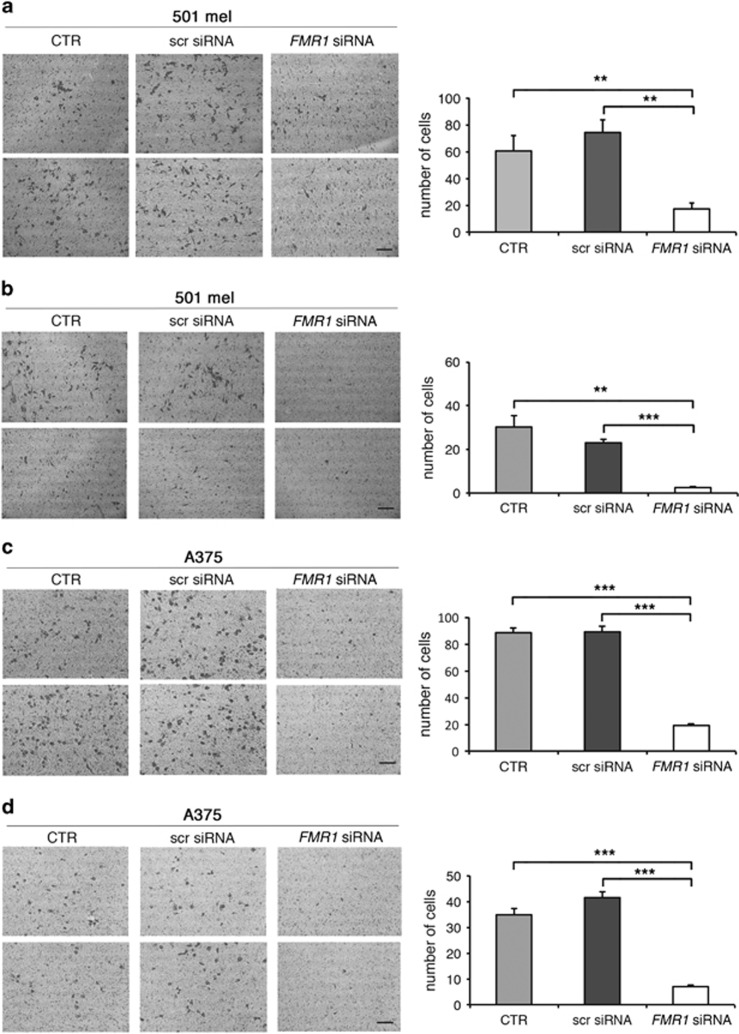
Downregulation of FMRP in melanoma cells affects migration and invasion. (**a**, Left panel) 501 mel cells were transfected with *FMR1* siRNAs or a scrambled siRNA (scr siRNA) or untransfected cells (CTR) and subjected to transwell cell migration assays 72 h post-transfection. Migrated cells were stained with crystal violet and counted at 6 h. Two representative pictures of migrating 501 mel cells are shown for each condition. (Right panel) Quantification; *n*=3, **P<0.01, Student's *t*-test. (**b**, Left panel) 501 mel cells were transfected with specific *FMR1* siRNAs or a scrambled siRNA (scr siRNA) or untransfected cells (CTR) and then subjected to Matrigel-coated transwell invasion assays 72 h post-transfection. Invading cells were stained with crystal violet and counted at 12 h. Two representative pictures of invading 501 mel cells are shown for each condition. (Right panel) Quantification; **P< 0.01, ***P<0.001, Student's *t*-test. (**c**, Left panel) A375 cells were transfected with *FMR1* siRNAs or a scrambled siRNA (scr siRNA) or untransfected cells (CTR) and subjected to transwell cell migration assays 72 h post-transfection. Migrated cells were stained with crystal violet and counted at 6 h. (Right panel) Quantification; *n*=3, ***P<0.001, Student's *t*-test. (**d**) A375 cells as in (**c**) using Matrigel-coated transwells. Invasion assay 72 h post-transfection. Invading cells were stained with crystal violet and counted at 12 h. (Right panel) Quantification; *n*=3, ***P<0.001, Student's *t*-test. Calibration bars, 250 *μ*m in all panels

After FMRP silencing, migration of 501 mel or A375 cells was followed for 9 h in a wound-healing assay (Figures 3a and b). Reduction of FMRP significantly inhibited the migration properties of both cell lines with respect to untransfected cells (control (CTR)) (Figures 3a and b). On the contrary, cells transfected with a scrambled siRNA were able to close the wound in a similar manner as the untransfected cells (Figures 3a and b). Cell migration was further evaluated using the transwell assay. The 501 mel showed a decreased migration upon FMRP silencing (Figure 4a), and A375 cells showed a similar trend (Figure 4c). We next investigated the invasion property of these two melanoma cell types using a Matrigel-coated transwell. Both melanoma cell types depleted of FMRP showed a reduced propensity to degrade the Matrigel and thus migrate through the membrane pores (Figures 4b and d). The survival rate of both melanoma cell lines in the three experimental conditions (CTR, scr siRNA or *FMR1* siRNA) did not change, excluding an important effect of FMRP on proliferation and/or cell death (Supplementary Figure 2).

**Figure 5:**
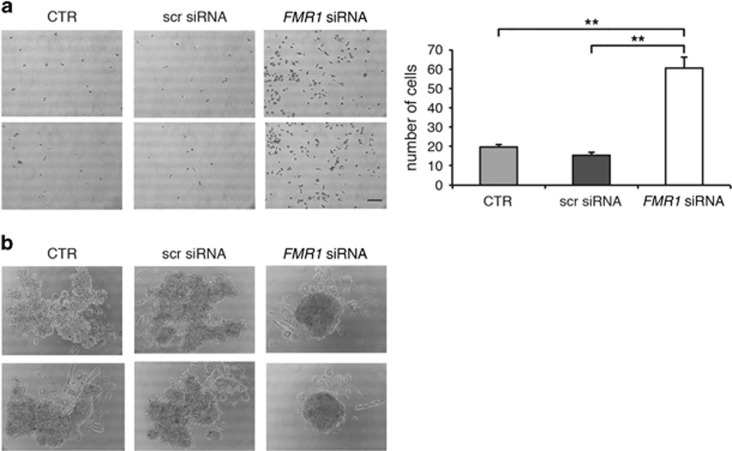
Downregulation of FMRP in melanoma cells affects cell–surface and cell–cell adhesion. (**a**, Left panel) A375 cells were transfected with specific *FMR1* siRNAs or a scrambled siRNA (scr siRNA) or untransfected cells (CTR) and 72 h post-transfection cells were plated for the adhesion assay. After 5 h, adherent cells were stained with crystal violet. Two representative pictures of adherent A375 cells are shown for each condition. (Right) Quantification; *n*=3, **P<0.01, Student's *t*-test. Calibration bar, 250 *μ*m. (**b**) Photographs of representative spheroids from 501 mel cells transfected with *FMR1* siRNAs, with a scrambled siRNA (scr siRNA) or untransfected (CTR)

Finally, adhesive properties of silenced cells were monitored with two different approaches. Using a 2D adhesion assay,^18^ cells silenced for FMRP exhibited a significant increase in adhesion to the bottom of the plate (Figure 5a and Supplementary Figure 3). Furthermore, when 501 mel cells were cultured as 3D spheroids,^36, 37^ an increased cell–cell adhesion capability, seen as a spheroid-like growth, was observed in *FMR1*-silenced cells (Figure 5b).

Taken together, these results indicate that FMRP knockdown significantly affects migration, invasion and adhesion properties of melanoma cells, suggesting a role for FMRP in tumor cell invasiveness.

### FMRP is localized at the leading edges of melanoma cells

**Figure 6:**
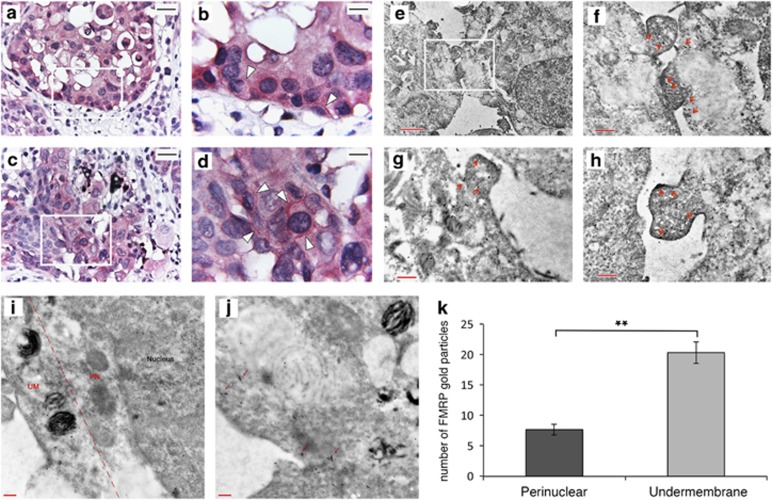
FMRP is localized at the leading edges of melanoma cells. (**a-d**) Representative immunohistochemical (IHC) images detecting FMRP expression in the invasive front of melanoma (a and c panels, × 400 magnification). Panel b, × 1000 magnification of the white box in panel a. Panel d, × 1000 magnification of the white box in panel c. The positivity pattern of melanoma cells is dotted, with linear gathering UM (b and d panels, see arrows). To differentiate the aspecific melanin signal from the specific FMRP immunopositivity, melanin was counterstained with Azur B (the asterisks mark the melanophages). (**e-h**) Representative electronic micrographics of IHC immunogold for FMRP in 501 mel cells. Panel e, × 5800 magnification of a cellular portion. Panel f, × 13 500 magnification of the white box showed in panel e. Panels g and h, × 13 500 magnifications of podosome structures localized at the leading edges of melanoma cells with FMRP-gold particles under the membrane region (red arrows in panels f-h). (**i, j**) Two representative immunogold EM images showing FMRP-gold particles in PN and UM regions in 501 mel melanoma cells (× 20 000 magnification). (**k**) Histogram showing the quantification of 5 fields of × 20 000 magnification *n*=20, **P<0.01, Student's *t*-test

Intracellular distribution of FMRP in human melanoma samples was first analyzed by IHC. A dotted pattern was gathered under the plasma membrane of melanoma cells (Figure 6b and d, white arrows). This pattern was further confirmed in 501 mel cells by IHC immunogold electron microscopy. Gold particles corresponding to FMRP were mainly localized in the cytoplasm and particularly concentrated under the plasma membrane and at the leading edges of the tumor cells (Figure 6f and h, red arrows). A quantification of FMRP-gold particles in perinuclear (PN) and under membrane (UM) area (× 20 000 magnification) of 501 mel cells showed that FMRP-gold particles were enriched in the region underlying the plasma membrane (Figure 6i and k) and often aggregated forming multicomponent complexes (Figure 6j, red arrows). Our findings suggest a role of FMRP in regulating mRNA metabolism at the leading edges in melanoma cells.

### FMRP regulates pathways involved in melanoma progression

**Figure 7:**
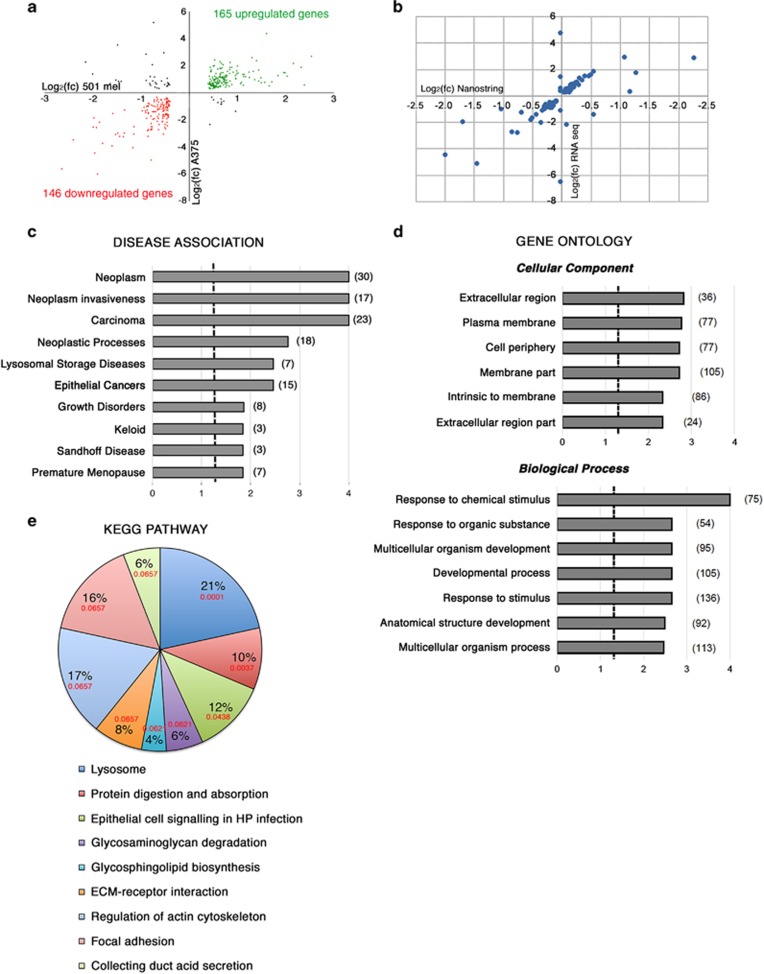
FMRP-regulated transcriptome and pathway analysis. (**a**) Deregulated mRNAs in melanoma cells. A correlation plot showing the 352 FRGs whose expression consistently and significantly changed in FMRP-depleted cells with respect to control cells, in both cell lines (501 mel and A375). Colored circles indicate the 311 FRGs whose expression consistently and significantly changed in both cell lines and in the same direction. The downregulated FRGs are shown in red and the upregulated FRGs are in green. The 41 FRGs whose expression changed in both cell lines but in the opposite directions. *n*=3 for both 501 mel and A375 are depicted in black. (**b**) Validation of the transcriptomic analysis on a subgroup of 91 annotated genes using the Nanostring nCounter (Nanostring Technologies, Seattle, WA, USA). A correlation plot for the 91 genes present in both RNA-seq gene set and Nanostring nCounter gene set. Ninety-three percent of the data points were present in the same dial of the graph (i.e. change in the same direction). (**c**) DEA of 311 more relevant FRGs showing the mRNA enriched in different disease categories. Brackets indicate the number of genes in each category. The x-axis represents the Log_10_ of *P*-value adjusted by the multiple test (adj*P*), and the dashed line indicates the significance threshold (Log_10_(adj*P*)=1.3). (**d**) Pathway analysis of 311 FRGs, using Gene Ontology (GO) functional annotation of Web Gestalt bioinformatics database. Partial diagram of ‘cellular component’ and ‘biological process’ categories, with the most significantly affected pathways. Brackets indicate the number of genes in each category. The x-axis represents Log_10_(adj*P*), and the dashed line the significance threshold (Log_10_(adj*P*)=1.3). (**e**) KEGG analysis. Pathways analysis of 311 FRGs, using KEGG functional annotation of Web Gestalt bioinformatics database. A pie chart of more representative pathways affected in KEGG functional annotation. In red, the adj*P* value are indicated for each category

RNA-seq technology was used to identify mRNAs with different expression levels in the absence of FMRP (named FMRP-Regulated Genes or FRGs) in melanoma. A comparative analysis of the transcriptome of two FMRP-depleted melanoma cells (*FMR1* siRNA; Supplementary Figure 4) *versus* CTR (Supplementary Figure 4) was performed. Analysis of the annotated human genes (25 312 genes) revealed that 10 205 were expressed in 501 mel and 3701 were expressed in A375 cells. Comparative analysis revealed that 5721 genes changed in 501 mel cells, 666 genes changed in the A375 cells and 352 genes changed consistently and significantly in both cell lines. Of these 352 genes, 311 changed consistently, significantly and in the same direction in both cell lines (Supplementary Table 1), whereas 41 genes changed in both cell lines but in the opposite directions. Interestingly, of the 311 more relevant FRGs, 165 genes (i.e. 52.7%) were upregulated and 146 (i.e. 47.3%) were downregulated (Figure 7a). A validation of the transcriptomic analysis was performed on a subgroup of 91 annotated genes using the Nanostring nCounter approach, a method that avoids sample amplification (http://www.nanostring.com). Of note, the RNA-seq data set showed a good correlation with the Nanostring nCounter (Pearson’s coefficient, 0.70) (Figure 7b).

Pathway analysis of the 311 more relevant FRGs using Web Gestalt bioinformatics database (http://bioinfo.vanderbilt.edu/webgestalt/) showed that the dysregulated genes were associated with diverse groups of biological processes and pathways. A Disease Enrichment Analysis (DEA) showed that FRGs were enriched in ‘neoplasms’, ‘neoplasm invasiveness’, ‘carcinoma’, ‘neoplastic process’ and ‘epithelial cancer’ categories (Figure 7c and Supplementary Table 1). This evidence confirms the involvement of FMRP in melanoma progression.

The Gene Ontology (GO) functional annotation showed that FRGs were related to different biological processes involved in invasive phenotype in tumor cells. In particular, the most significantly affected pathways were strictly related to FMRP functions in breast cancer cells^18^ and in neurons.^5, 38^ The GO analysis suggests that one of the FMRP functions might be to regulate proteins belonging to periphery/plasma membrane/extracellular pathways involved in cancer cell invasiveness (Figure 7d). Of the 419 reference genes in the ‘extracellular region’ category, 38 were present in the FRGs gene set, against an expected number of 18.48 (*R*=2.06; raw*P*=1.27e−05; adj*P*=0.0015). Furthermore, within the ‘biological processes’ category of GO annotation, the most significantly affected pathway was ‘response to chemical stimulus’ (Figure 7d and Supplementary Figure 5). Seventy-five genes of the FRG set were found in this group against an expected number of 42.16 (*R*=1.78; raw*P*=9.52e−08; adj*P*=0.0001), supporting a role of FMRP in regulating cancer cellular dynamics in response to different chemical stimuli (paracrine and/or endocrine) that occur during migration and invasion.^39^

Finally, the analysis in the KEGG pathway database showed that the FMRP-affected proteins were part of pathways involved in peripheral cell plasticity during migration and invasion processes, such as ‘regulation of actin cytoskeleton’ and ‘focal adhesion’ (Figure 7e). Intriguingly, 11 FRGs were present in the ‘lysosome’ category, against an expected number of 1.88, and therefore with an enrichment of 5.84 fold changes (raw*P*=1.62e−06; adj*P*=0.0001).

Similar results for GO and KEGG pathway analysis were also obtained using DAVID online bioinformatic database (https://david.ncifcrf.gov) (data not shown).

## Discussion

Owing to a rapid systemic dissemination and high capacity of metastatization, melanoma is a highly aggressive neoplasm^24, 25^ that is extremely refractory to conventional antineoplastic treatments.^40, 41^

Although there are hundreds of studies that have sought to assess the potential prognostic value of molecular markers in predicting the course of cutaneous melanoma, at this time, no molecular method to improve risk stratification is part of recommended clinical practice.^42^ The identification of molecular biomarkers in melanoma is therefore of utmost importance.

FMRP has been largely studied in the brain,^43^ where it has an important role in the local regulation of mRNA metabolism, and only recently in breast cancer.^18^ Despite the fact that neurons, breast cancer cells and melanoma cells share a number of similarities in their gene expression pattern,^44, 45, 46^ signaling pathways (including PKC- and p53/p73-dependent pathways) and signaling molecules (such as Wnt, fibroblast growth factors and neurotrophins),^44^ FMRP function/s in melanocytic cells or in their tumoral counterpart has never been investigated.

Our work revealed that FMRP is overexpressed in melanoma cells with respect to normal melanocytes (Figures 1 and 2) and its expression significantly correlates with two main prognostic factors of melanoma cancer progression: Breslow thickness (how deeply tumor cells have invaded) and Clark level (level of anatomical invasion of the melanoma in the skin); furthermore, the analysis of *FMR1* expression levels in the cohort included in the melanoma TCGA skin cutaneous melanoma indicates that FMRP expression correlates with the risk of tumor relapse and disease-free survival (Figure 1). These data show for the first time that FMRP is involved in cutaneous melanoma progression and suggest a conserved function for FMRP in melanoma and neuronal cells. This hypothesis was confirmed by the observation that the reduction of FMRP levels in two different melanoma cell lines (501 mel and A375) affects their invasion, migration and adhesion (Figures 3, 4, 5) – processes that have a central role in metastatization.^47, 48^

It is well established that mRNA localization and local protein synthesis largely contribute to cancer progression and metastatization^49, 50, 51^ by assisting in the establishment and maintenance of cancer cell polarity and behavior plasticity during migration and invasion.^52, 53^ Indeed, in polarized invading cells, there is an asymmetric distribution of many cytoskeletal and signaling proteins, as well as some mRNAs whose stability/translation are locally regulated by specific RBPs for establishing and maintaining front–rear polarity and directional cell migration.^54^ Converging evidence over the past years suggest that FMRP may represent one of these crucial RBPs. Indeed, in neurons, FMRP is part of large ribonucleoprotein (RNP) complexes that contain motor proteins such as kinesin KIF3C, dynein and myosin Va,^11, 55, 56, 57^ and show a typical peripheral localization and gathering under the surface membrane of neurons, as well as in the proximity of mGluR receptors in the postsynaptic dendritic spines.^29^ Similarly, in fibroblasts, FMRP was enriched in granules containing translationally silent mRNAs, localized in the peripheral parts of the cells and in protruding pseudopodia of migrating fibroblasts.^58^

Here we show for the first time that FMRP has a similar localization in tumor cells, enriched with a punctate staining pattern below the plasma membrane in specific pseudopodia-like cellular protrusions (Figure 6). Similarly, FMRP was detected in transport granules via myosin Va in B16-F10 murine melanoma cell lines.^59^ Altogether, these data strongly suggest that FMRP is able to regulate RNP complexes in both polarized neuronal and cancer cells.

Of note, using an NGS transcriptome approach, we identified a wide panel of mRNAs with altered levels in the absence of FMRP (named FRGs) (Figure 7a and Supplementary Table 1) that confirms a role for FMRP in regulating (directly or indirectly) processes that occur in the peripheral part of the cells and that contribute to the modulation of the cytoskeletal and morphological plasticity at the leading edges of cancer cells during migration and invasion (Figures 7c and d). In particular, FMRP is able to modulate the expression of several mRNAs encoding proteins capable of remodeling the extracellular matrix such as matrix metalloproteinase-8, MMP8, the disintegrins and metalloproteases ADAM19 and ADAM23 and the metalloprotease inhibitor TIMP2 (Supplementary Table 1). Accumulated evidence emphasize the importance of the tumor microenvironment in enhancing the aggressive behavior of melanoma cells.^60^ Several proteolytic enzymes, such as cysteine proteases, MMPs and the ADAMs, were shown to be pivotal in promoting melanoma cell invasion. These enzymes not only remodel the extracellular matrix but also release active factors and shed cell surface receptors, thereby mediating melanoma cross-communication with their microenvironment. The exact players and mechanisms that enable a tumor to activate the tumor microenvironment are not completely understood, but it is well known that melanoma cells engineered to express the *BRAF*-mutated form, *BRAF*^V600E^, expressed higher levels of the secreted proteins, IL-1*β*, IL-6, IL-8 and MMP-1, than wild-type cellular counterparts.^61^

Interestingly, FMRP appears to also regulate the expression of MAPK pathway-related mRNAs (i.e. MAPK4) (Supplementary Table 1) that have a key role in the development of melanoma.^62^ However, further studies are required to investigate the exact role of these molecules in FMRP-overexpressing melanoma cells. Another well-represented pathway in the KEGG database is the ‘lysosome’ pathway. These data are particularly interesting because endolysosomal trafficking in melanoma is hyperactivated and particularly distinguishes this disease from over 35 different cancer types.^63^ As melanoma cells are revealing intrinsic vulnerabilities of the endolysosomal machinery, this pathway can be harnessed for tumor-selective drug delivery and cell death.^64, 65^

In conclusion, FMRP regulates molecular processes that impact upon the ability of tumor cells to interact with their environment and switch from a sessile, stationary to a migratory and invasive phenotype. It is therefore tempting to propose that FMRP levels could aid in the identification of high-risk melanoma patients at the time of original diagnosis, contributing significantly to improved patient outcomes and increased survival. Additionally, anticancer therapies that modulate FMRP levels or FMRP-related genes relevant to the biology of the invasive melanoma could represent a promising option for treatment.

Finally, RBPs have been proposed as key molecular links between cancer and neurological disorders, two processes that, despite that it seemingly has little in common, have been found to be significantly associated in a wide number of epidemiological studies.^66^ Since understanding the association between cancer and neurological disorders constitutes a fascinating approach to obtaining clues to underlying the pathogenesis of both conditions, our data may open major avenues to the development of future therapeutic strategies in both melanoma and FXS.

## Materials and methods

### Immunohistochemical analysis

FMRP-IHC was performed using polyclonal antibodies^29^ (1 : 50 dilution), followed by detection with labeled polymer in accordance with the standard UltraVision AP detection system (Thermo Fisher Scientific, Runcorn, UK). Immunohistochemical reactions were visualized by using Liquid Fast-Red Substrate System (Thermo Fisher Scientific) as the chromogen. Hematoxylin and Azure B were used as counterstains to visualize nuclei and melanin, respectively. A semiquantitative approach was used to evaluate FMRP protein expression. FMRP expression was quantified as the product between a score for the extent and for the intensity of staining positivity. The percentage of positive cells was measured in 200x field (7-10) randomly chosen and expressed as a mean percentage for each sample. A criterion value of 35% was calculated by an ROC curve and a score of 0–1 was derived as follows: 0, ≤35% positive cells and 1, >35% of them. The intensity of staining was recorded on a scale of 0–1, in which 0=negative or weakly positive and 1=moderately or strongly positive. Two researchers blind to the patients’ data, using a double-headed microscope, independently performed the immunohistochemical evaluation. Intraobserver agreement was higher than 90%. These findings were confirmed on patients recruited in two different hospitals.

### Tumor cell lines

Primary NHEMs and human adult melanocytes were purchased from Lonza (Basel, Switzerland) and maintained in MGM-4 medium with supplemented growth factors from Lonza. The MM001, MM011, MM031, MM032, MM034, MM047, MM057, MM074, MM087, MM099, MM117 and MM118 melanoma cell lines were derived from patients with invasive and metastatic melanomas, upon being shortly cultured in F10 medium with 5% fetal bovine serum (FBS; (HyClone Laboratories, UT, USA) and 5% calf bovine serum (HyClone Laboratories).^30^ The human melanoma cell line 501 mel were established from metastases obtained from melanoma patients surgically resected at the Istituto Nazionale dei Tumori (Milan, Italy) and maintained as described.^67^ The human melanoma cell line A375 was generously provided by P Arcidiacono and A Cristante from the Department of Experimental Medicine (University of Perugia, Perugia, Italy) and maintained as described.^68^

### Meta-analysis of TCGA

*FMR1* expression data (RNA-seq V2) and clinical and pathological information of a cohort of 472 patients with skin cutaneous melanoma (TCGA, provisional) was downloaded from cBioPortal website (http://www.cbioportal.org/). Exclusion criteria were applied to the cohort, that is: (i) patients who received neoadjuvant treatment before sample collection; (ii) metastatic melanoma with an unknown origin; (iii) secondary metastatic melanoma. In total, 402 out of 472 samples were retained for all analyses described herein. *FMR1* RPKM-level data were log 2 transformed. For the survival analysis, samples with an *FMR1* expression lower than the 25th percentile of the distribution were assigned to the LOW class, whereas patients with expression higher than 75th percentile were assigned to the HIGH class. JMP 12 (SAS Institute Inc, Cary, NC, USA) software was used for all statistical analyses including Kaplan–Meier survival analysis.

### Silencing of *FMR1* mRNA using siRNAs

siRNA-mediated silencing of FMRP were performed with *FMR1*-specific siRNAs from Life Technologies (Carlsbad, CA, USA) (AM 16708, ID nos 10824, 10919 and 11010). As a nonspecific control, a scrambled siRNA was used (no. 4390843; Life Technologies). siRNA duplex was transfected into melanoma cells (501 mel or A375) using Lipofectamine RNAiMAX (Life Technologies), according to the manufacturer’s instructions. Transfections were carried out in six-well plates at 50–60% confluency with 90 pmol of siRNA, and cells were harvested after 24, 48 or 72 h.

### Wound-healing assay

For assessing the migratory ability in a scratch assay, 7 × 10^4^ melanoma cells (501 mel or A375) were transfected with *FMR1*-specific siRNAs or a scrambled siRNA or untransfected cells (CTR) and were harvested to silence FMRP expression. After 72 h, the cell monolayer was scratched using a pipette tip through the central axis of the plate. Migration of the cells into the scratch was digitally documented 0, 3, 6 and 9 h after being made, and relative migratory activity was calculated based on the cell-free areas.

### Transwell migration or invasion assay

For the migration assay, melanoma cells (501 mel or A375) transfected with *FMR1*-specific siRNAs, or a scrambled siRNA or untransfected cells (CTR), were starved overnight in RPMI-1640 and only 2% FBS. Cells (7.5 × 10^4^) were added to the top chambers of 24-well transwell plates (Corning Costar, Kennebunk, ME, USA; 8 *μ*m pore size), and RPMI-1640 media with 20% FBS was added to the bottom chambers. After overnight incubation, top (non-migrated) cells were removed and bottom (migrated) cells were fixed with 4% paraformaldehyde and stained with 0.1% crystal violet (5 mg/ml in 2% ethanol from Sigma, St. Louis, MO, USA) for 40 min at room temperature. Cells remaining on the upper side of the filter were removed with a cotton swab. The number of migrating cells in five fields was counted under 20x magnification, and the mean for each chamber determined. Experiments were repeated a minimum of three times.

For the invasion assay, Matrigel-coated transwells containing 8 *μ*m pores (Corning Costar) were used. Cells (7.5 × 10^4^) were seeded into the upper chamber in RPMI-1640 medium with 2% FBS and media with 20% FBS were added to the lower chamber. Cells were fixed in 4% paraformaldehyde 24 h later and stained with 0.1% crystal violet. Invading cells were quantified as for migration assays.

### MTT assay

Melanoma cells (501 mel or A375) were transfected with *FMR1*-specific siRNAs, or a scrambled siRNA or untransfected cells (CTR), and 72 h after transfection, cells were seeded on 96-well microplates (1 × 10^5^ cells per well) and 10 *μ*l of MTT reagent (Biotium, Fremont, CA, USA) were added in each well and incubated at 37 °C. After 2, 6 or 12 h, cell viability was revealed through the conversion of the water-soluble MTT to insoluble formazan. Formazan was solubilized adding 200 *μ*l of dimethylsulfoxide and its concentration was measured by optical density at 570 nm.

### Adhesion assay

A suspension of 500 *μ*l of RPMI-1640 medium with 2% FBS, containing 1.7 × 10^3^/ml 501 mel or A375 cells was plated in each well of a 24-well plate for 5 h. The plates were washed with DPBS 1 × and then fixed with 96% ethanol for 15 min at room temperature. The cells were washed and stained with 0.1% crystal violet for 40 min. The number of adherent cells in five fields was counted under 20x magnification, and the mean for each chamber determined. Experiments were repeated a minimum of three times.

### 3D multicellular tumor spheroid adhesion assay

The 501 mel confluent cultures were trypsinized, washed in DPBS and resuspended in DMEM-F12. Cells/spheroid (5 × 10^6^) were suspended in 20% methylcellulose in DMEM-F12 plus EGF. Drops of the cell suspension were placed onto the lid of 150 mm dish, which was then flipped over the dish. Hanging drop cultures were incubated for 24 h and the resulting cellular aggregates were washed and recovered with a large pipette tip and harvested according to Hattermann *et al.*^37^

### Western blotting

Cells were lysed in 100 mM NaCl, 10 mM MgCl_2_, 10 mM Tris-HCl (pH 7.5), 1% Triton X-100, 1 mM DTT, 40 U/ml RNAse OUT (Life Technologies, Carlsbad, CA, USA), 5 mM *β*-glycerophosphate, 0.5 mM Na_3_VO_4_, 10 *μ*l/ml Protease inhibitor cocktail (PIC; Sigma) or 50 mM Tris-HCl (pH 7.4), 150 mM NaCl, 1% DOC, 1% NP-40 and 10 *μ*l/ml PIC. After 5 min of incubation on ice, the lysates were centrifuged 15 min at 16 000 × *g* at 4 °C. Supernatants (10–20 *μ*g) were separated by SDS-PAGE electrophoresis and transferred to a PVDF membrane (Millipore, Carrigtwohill, County Cork, Ireland). Membranes were incubated using specific antibodies for FMRP^29^ (1 : 500), *β*-actin (1 : 1000; Cell Signaling (Leiden, The Netherlands)) and *α*-tubulin (1 : 5000; DSHB, Iowa City, IA, USA) and the signal was detected using an Enhanced Chemiluminescence Kit (GE Healthcare, Little Chalfont, UK). Membranes were then stained with Coomassie.

### Immunogold electron microscopy

501 mel and A375 cells lines were fixed for 3 h at 4 °C in a mixture of 2% paraformaldehyde and 0.1% glutaraldehyde in 0.1 M cacodylate buffer (pH 7.6). They were dehydrated in alcohol at progressively higher concentrations and embedded in Bioacryl resin (British Biocell, Cardiff, UK), followed by UV polymerization according to standard procedures. Ultrathin sections were cut and mounted on 300 mesh nickel grids. To block nonspecific binding sites, the grids were treated with a blocking buffer made of phosphate-buffered saline supplemented with 0.1% Tween-20, 0.1% bovine serum albumin and 4% normal goat serum. FMRP antibodies was used at a 1 : 25 dilution. For single localization experiments, grids were incubated overnight in the presence of FMRP primary antibodies. The grids were incubated for 1 h with goat anti-rabbit IgG conjugated with 15 nm colloidal gold particles (British Biocell). Sections were then counterstained in uranyl acetate to display cell morphology and observed under vacuum with an EM TECNAI G2 microscope (FEI Company, Hillsboro, OR, USA). Photographs were taken with a digital camera Multi Scan 2 K × 2 K.

### RNA-sequence analysis

Total RNA was extracted from control or *FMR1*-silenced 1 × 10^6^ cells (501 mel and A375) using Trizol reagent (Life Technologies). Three independent silencing experiments were performed for both 501 mel and A375 cells (Supplementary Figure 4). The quality of the RNA was verified on a Bioanalyser (Agilent, Santa Clara, CA, USA). Libraries were constructed using the TruSeq RNA Sample Kit (Illumina, San Diego, CA, USA) according to the manufacturer’s protocol. Final libraries were pooled and sequenced on the HiSeq 2000 (Illumina), generating a total of 220 million reads of 50 bp length. Reads were mapped to the human reference genome (hg18) using TopHat v1.3.3 (PMID: 19289445) with default settings. Mapped reads were assigned to genes using the human RefSeq annotation and counted using HT-Seq. Normalization and differential expression analyses were performed using DESeq (PMID: 20979621). All genes were selected for further analysis that showed – in both cell lines – a different expression between mock and *FMR1*-silenced samples with an adjusted *P*-value of <0.25.

### Functional annotation

The functional analysis of DEGs was performed using the WEB GESTALT tool (http://bioinfo.vanderbilt.edu/webgestalt) and DAVID tool (http://david.abcc.ncifcrf.gov). Total transcriptome of 501 mel and A375 melanoma cells was used as the background list for the over-representation analysis. The GO option was used, and the significantly (*P*<0.05) enriched biological processes and groups of genes possibly contributing to FMRP-dependent gene expression regulation were identified. The KEGG (http://www.genome.jp/kegg) was used to identify pathways that were most significant to the data set.

### Nanostring nCounter analysis

Hundred nanograms of total RNAs (previously RNA-seq analyzed) from 501 mel cells were subjected to Nanostring nCounter analysis, using the GX Human Cancer Reference Kit (Nanostring Technologies (Seattle, WA, USA); no. GXA-CR1-12). Among the 230 genes of this GX Kit, 91 overlap with the RNA-seq gene set. Nanostring nCounter analysis was performed following the manufacturer’s protocol. Raw counts for each mRNA were normalized and analyzed using nSolver Analysis Software (Nanostring Technologies) to obtain differential expression analysis of 91 genes in *FMR1*-depleted cells *versus* WT cells.

### Human tissues collection and patient information

The studies described in this paper that involve human samples have all been performed with informed consent from the patients. Human melanomas and cells from patients used in this study were provided by Pathological Anatomy Units of Campus Bio-Medico University (Rome, Italy) and by the University of Turin (Turin, Italy). All experiments involving human specimens were conformed to the principles described in the NMA Declaration of Helsinki and the NIH Belmont report. The histopathological diagnoses of the tumors were described according to the World Health Organization International Classification of Disease for Oncology. The clinical staging was determined in agreement with the AJCC 2009 staging system.^69^ All human tissues were collected following standardized procedures and informed consent was obtained for all specimens linked with clinical data according to procedures approved by the Institutional Ethical Board of the European Institute of Oncology. Each sample was histopathologically evaluated to ensure the presence of at least 80% of tumor cells. The medical records of all patients were examined to obtain clinical and histopathological information.

## Publisher’s Note

Springer Nature remains neutral with regard to jurisdictional claims in published maps and institutional affiliations.
