# Supplementary material for: The fragile X mental retardation protein regulates tumor invasiveness-related pathways in melanoma cells
Source: Cell Death Dis. 2017 Nov 16;8(11):e3169–. doi: 10.1038/cddis.2017.521 (PMC5775405; doi:10.1038/cddis.2017.521)
Supplement: Supplementary Information [file cddis2017521x1.pdf]

Supplementary Information

The Fragile X Mental Retardation Protein regulates tumor invasiveness-related pathways in melanoma cells

Francesca Zalfa, Vincenzo Panasiti, Simone Carotti, Maria Zingariello, Giuseppe Perrone, Laura Sancillo, Laura Pacini, Flavie Luciani, Vincenzo Roberti, Silvia D'Amico, Rosa Coppola, Simona Osella Abate, Rosa Alba Rana, Anastasia De Luca, Mark Fiers, Valentina Melocchi, Fabrizio Bianchi, Maria Giulia Farace, Tilmann Achsel, Jean-Christophe Marine, Sergio Morini and Claudia Bagni.

Supplementary Figures & Legends

Supplementary Figure 1

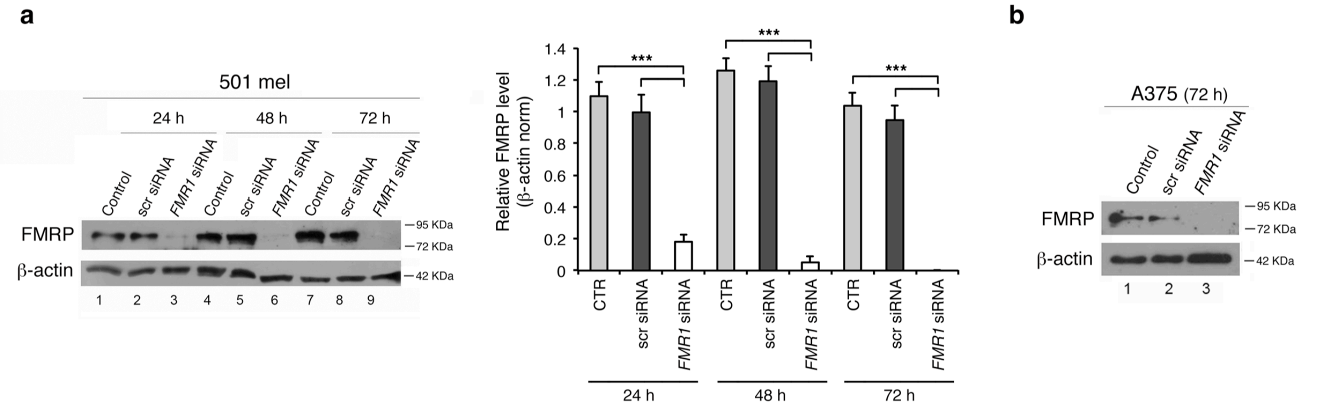

Supplementary Figure 1. FMRP levels were detected by western blotting after 24, 48 and 72 h of *FMR1* silencing in 501 mel cells **(a)** and A375 cells **(b).** Note that only the 72 h time point is shown for the A375 cells.

Supplementary Figure 2

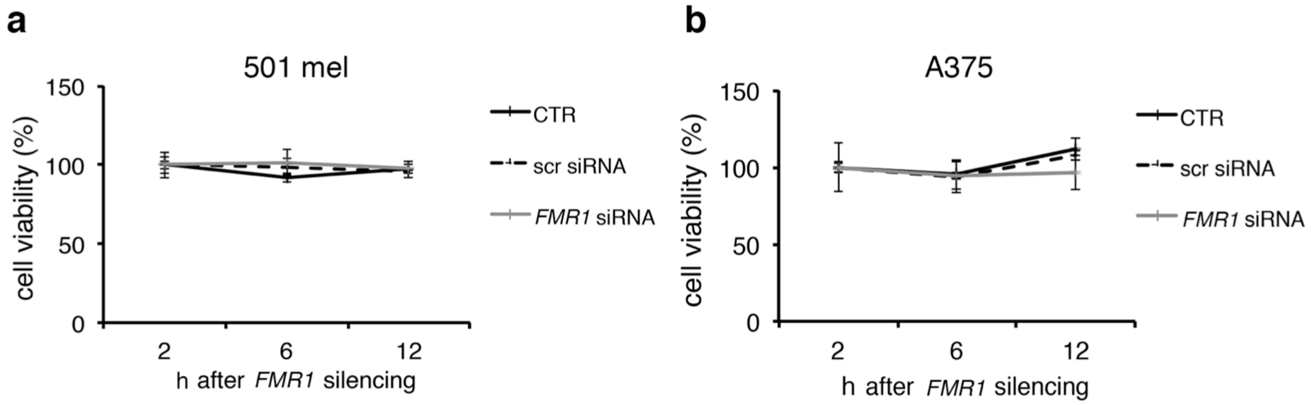

Supplementary Figure 2. 501 mel cells (a) or A375 cells (b) untransfected (CTR) or transfected with specific *FMR1* siRNAs or a scrambled siRNA (scr siRNA). After 2, 6 or 12 h, cell viability was measured with an MTT assay. $n = 3$, $p > 0.05$, Student's *t* test, for both cell lines and experimental conditions.

Supplementary Figure 3

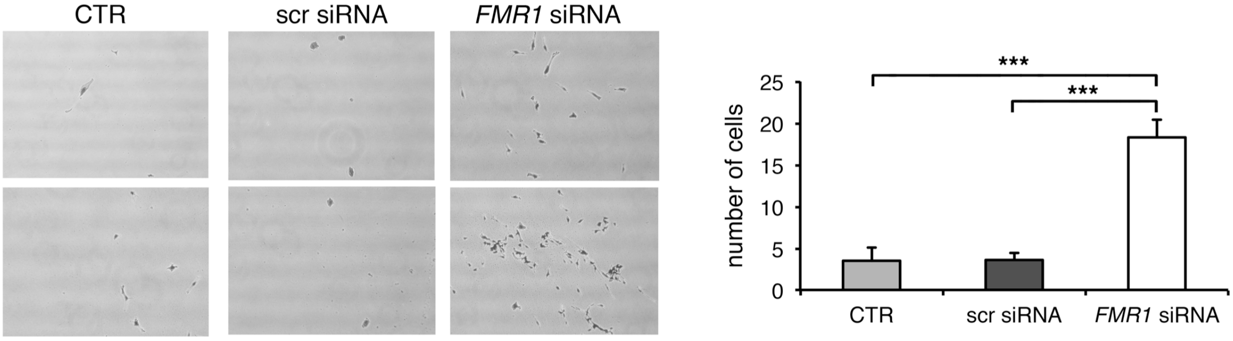

Supplementary Figure 3. 501 mel cells were transfected with specific *FMR1* siRNAs or a scrambled siRNA (scr siRNA) or untransfected (CTR) and 72 h post-transfection cells were plated for a adhesion assay. After 5 h, adherent cells were stained with crystal violet. n = 3, ***$p < 0.001$, Student's *t* test.

Supplementary Figure 4

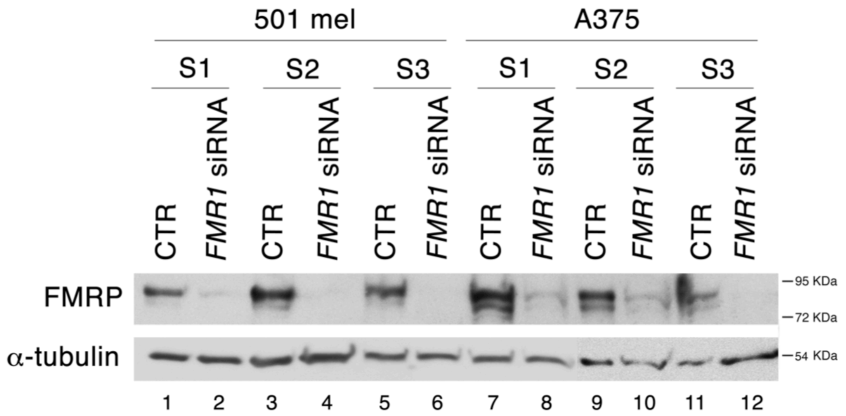

Supplementary Figure 4. Representative western blot of FMRP and α-tubulin of the three independent cultures of *FMR1*-silenced cells (501 mel and A375 cells) used for RNA sequence analysis.

Supplementary Figure 5

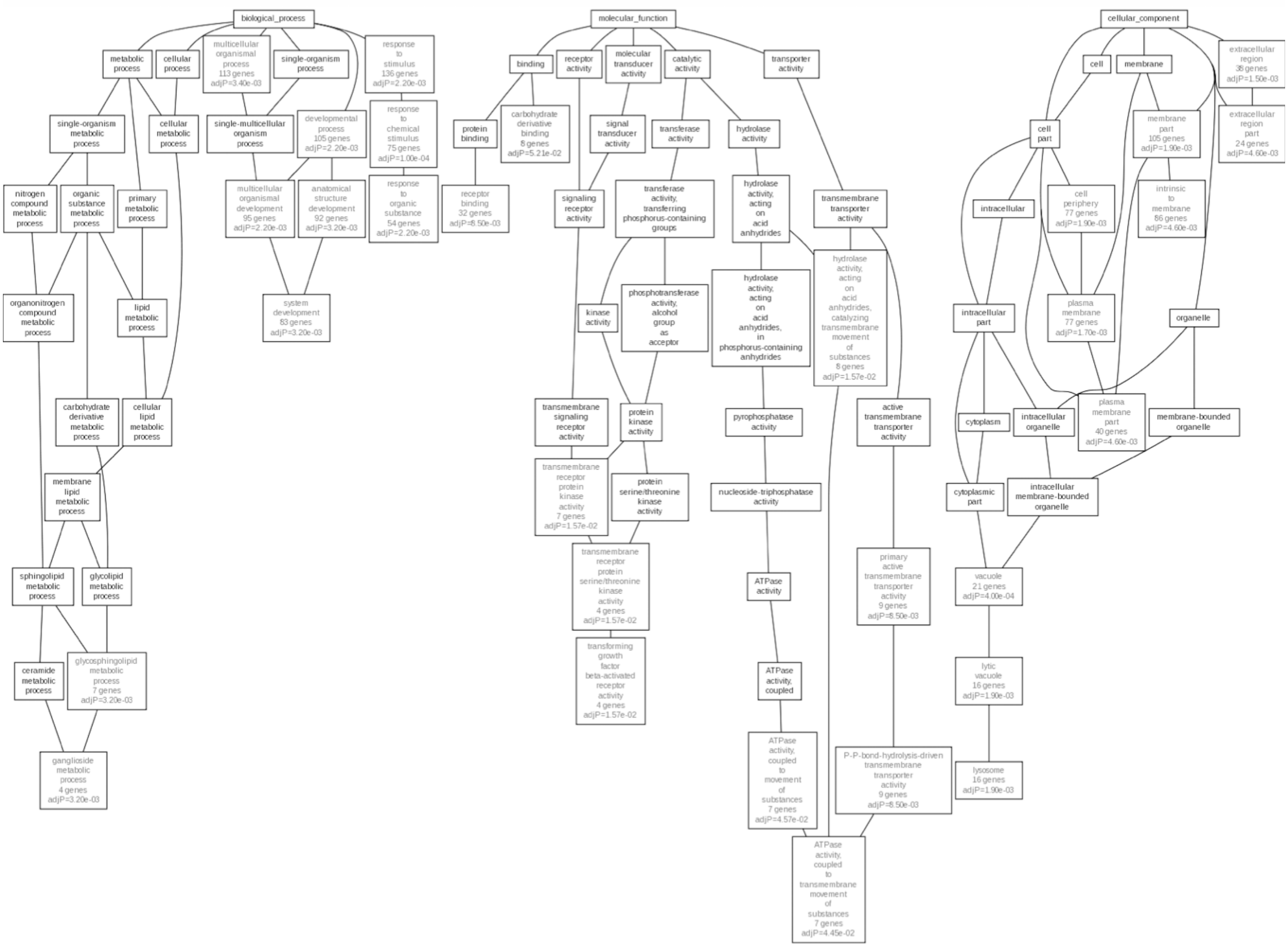

Supplementary Figure 5: Complete diagram of pathway analysis of 311 FMRP Regulated Genes (FRGs), using GO functional annotation of Web Gestalt bio-informatics database.

Supplementary Tables

Supplementary Table 1. List of 311 FMRP-Regulated Genes (FRGs) in both 501 mel and A375 melanoma cells * (see Excel file)

* For each gene is reported: Gene symbol, Gene name, Log_{10}(fold change), signed Log_{10}(P) (SLP) and the presence in the top three most significant categories of Disease Association Analysis, GO Analysis and KEGG pathway analysis respectively.

For DISEASE ASSOCIATION the following categories are reported: Neoplasm (C = 270; O = 30; E = 11.30; R = 2.66; rawP = 7.66e-07; adjP = 0.0001); Neoplasm Invasiveness (C = 99; O = 17; E = 4.14; R = 4.10; rawP = 5.75e-07; adjP = 0.0001); Carcinoma (C = 174; O = 23; E = 7.28; R = 3.16; rawP = 7.98e-07; adjP = 0.0001).

For BIOLOGICAL PROCESS of GO Analysis are reported: Response to chemical stimulus (C = 952; O = 75; E = 42.16; R = 1.78; rawP = 9.52e-08; adjP = 0.0001); Response to stimulus (C = 2297; O = 136; E = 101.72; R = 1.34; rawP = 4.62e-06; adjP = 0.0022); Multicellular organismal development (C = 1441; O = 95; E = 63.81; R = 1.49; rawP = 5.50e-06; adjP = 0.0022).

For MOLECULAR FUNCTION of GO Analysis are reported: Primary active transmembrane transporter activity (C=44; O = 9; E = 1.92; R = 4.68; rawP = 9.35e-05; adjP=0.0085); Receptor binding (C = 356; O = 32; E = 15.57; R = 2.06; rawP = 6.31e-05; adjP=0.0085); P-P-bond-hydrolysis-driven transmembrane transporter activity (C = 44; O = 9; E = 1.92; R = 4.68; rawP = 9.35e-05; adjP = 0.0085).

For CELLULAR COMPONENT of GO Analysis are reported: Vacuole (C = 148; O = 21; E = 6.53; R = 3.22; rawP = 1.67e-06; adjP = 0.0004); Extracellular region (C = 419; O = 38; E = 18.48; R = 2.06; rawP = 1.27e-05; adjP = 0.0015); Plasma membrane (C = 1128; O = 77; E = 49.74; R = 1.55; rawP = 2.23e-05; adjP = 0.0017).

For KEGG PATHWAY ANALYSIS are reported: Lysosome (C = 45; O = 11; E = 1.88; R = 5.84; rawP = 1.62e-06; adjP = 0.0001); Regulation of actin cytoskeleton (C = 84; O = 9; E = 3.51; R = 2.56; rawP = 0.0081; adjP = 0.0657); Focal adhesion (C = 73; O = 8; E = 3.05; R = 2.62; rawP = 0.0108; adjP = 0.0657).

C = the number of reference genes in the category; O = the number of genes in the gene set and also in the category; E = the expected number in the category; R = ratio of enrichment; rawP = p value from hypergeometric test; adjP = p value adjusted by the multiple test adjustment.
